# Emergence of NDM-1- and CTX-M-3-Producing *Raoultella ornithinolytica* in Human Gut Microbiota

**DOI:** 10.3389/fmicb.2019.02678

**Published:** 2019-11-22

**Authors:** Shuang Wang, Liuchen Xu, Xiaohui Chi, Yan Li, Zengqiang Kou, Peibin Hou, Hengjie Xie, Zhenwang Bi, Beiwen Zheng

**Affiliations:** ^1^Bacterial Infection Disease Control of Institute, Shandong Center for Disease Control and Prevention, Jinan, China; ^2^Collaborative Innovation Center for Diagnosis and Treatment of Infectious Diseases, State Key Laboratory for Diagnosis and Treatment of Infectious Diseases, The First Affiliated Hospital, College of Medicine, Zhejiang University, Hangzhou, China; ^3^Department of Environment and Health, School of Public Health, Shandong University, Jinan, China; ^4^Department of Supervise Sampling, Shandong Institute for Food and Drug Control, Jinan, China; ^5^Shandong Academy of Clinical Medicine, Shandong Provincial Hospital, Jinan, China

**Keywords:** *Raoultella ornithinolytica*, carbapenemase, extended-spectrum β-lactamase, *bla*_NDM-1_, *bla*_CTX-M_, whole-genome sequencing

## Abstract

*Raoultella ornithinolytica* is an opportunistic pathogen of the Enterobacteriaceae family and has been implicated in nosocomial infections in recent years. The aim of this study was to characterize a carbapenemase-producing *R. ornithinolytica* isolate and three extended-spectrum β-lactamase (ESBL)-producing *R. ornithinolytica* isolates from stool samples of adults in a rural area of Shandong Province, China. The species were identified using matrix-assisted laser desorption/ionization time-of-flight mass spectrometry (MALDI-TOF MS) and 16S rDNA sequence analysis. Antimicrobial susceptibility test showed that all four isolates were multidrug-resistant (MDR). The whole genome sequence (WGS) of these isolates was determined using an Illumina HiSeq platform, which revealed MDR-related genes. The S1 nuclease-pulsed-field gel electrophoresis (S1-PFGE) was used to characterize the plasmids carried by the *R. ornithinolytica* isolates. The *bla*_NDM-1_ and *bla*_CTX-M-3_ genes were probed using Southern blotting, which confirmed the location of both genes on the same plasmid with molecular weight of 336.5–398.4 kb. The transferability of *bla*_NDM-1_ and *bla*_CTX-M_ was also confirmed by conjugation assays. Finally, BLAST analysis of both genes showed that mobile genetic elements were associated with the spread of drug resistance genes. Taken together, we report the presence of conjugative *bla*_NDM-1_ and *bla*_CTX-M_ plasmids in *R. ornithinolytica* isolates from healthy humans, which indicate the possibility of inter-species transfer of drug resistance genes. To the best of our knowledge, this is the first study to isolate and characterize carbapenemase-producing *R. ornithinolytica* and ESBL-producing *R. ornithinolytica* isolates from healthy human hosts.

## Introduction

*Raoultella* is a genus of encapsulated Gram-negative aerobic bacilli of the Enterobacteriaceae family (Luo et al., 2017) that was initially part of the genus *Klebsiella*, but later reclassified based on the 16S rDNA sequence and the *rpoB*, *gyrA*, and *gyrB* genes (Drancourt et al., 2001). *Raoultella ornithinolytica* is one of the three species of the genus *Raoultella* (Singh et al., 2017) and naturally exists in the soil, water, and plants (Ayoade et al., 2018). *R. ornithinolytica* can cause pneumonia, biliary or urinary tract infections and bacteremia and such cases are being increasingly reported (Sekowska et al., 2015; Van Cleve et al., 2018). Both organ-specific and systemic *R. ornithinolytica* infections can be life-threatening, especially in cancer patients after abdominal surgery (Hajjar et al., 2018). Therefore, it is vital to understand the pathogenic potential of *R. ornithinolytica* isolates in humans (De Petris and Ruffini, 2018). Cephalosporins, quinolones, and carbapenems are routinely used against this pathogen, and multi-drug resistant (MDR) *R. ornithinolytica* isolates have been reported in recent years (Wang et al., 2019).

Widespread use of third-generation cephalosporins and other β-lactam antibiotics in the past decades have led to the emergence of third-generation cephalosporin-resistant bacteria that produce extended spectrum β-lactamases (ESBLs) and AmpC β-lactamases that are, respectively, encoded by plasmids and chromosomes (Kola et al., 2012; Dohmen et al., 2015). Consistent with this, infections caused by the ESBL-producing Gram-negative bacilli (GNB) have become increasingly prevalent worldwide, both in the healthcare and community settings, and pose significant therapeutic challenges (Wang et al., 2015). The class A serine CTX-M-type is the most common plasmid-encoded ESBLs that are produced by drug-resistant pathogens (He et al., 2016). The genetic elements encoding these CTX-M enzymes constantly evolve *via* random mutations and recombination between different resistance genes (Canton et al., 2012). A genetic epidemiological study on ESBLs found that *bla*_CTX-M_ has replaced *bla*_SHV_ and *bla*_TEM_ as the most common ESBL-encoding genes (Xia et al., 2014). While CTX-M-15 and CTX-M-14 are the most prevalent ESBLs worldwide (He et al., 2016), *bla*_CTX-M-14_ has been identified as the most prevalent ESBL gene in China, and epidemiological surveillance in Asia, Latin America, and Europe has revealed a dramatic increase in cephalosporin-resistant *Escherichia coli* and *Klebsiella* spp. strains due to spread of the CTX-M ESBLs (Zhang et al., 2016).

Carbapenem-resistant Enterobacteriaceae (CRE) is a serious public health concern worldwide because of its rapid spread and limited therapeutic drugs (Zheng et al., 2018). Metallo-β-lactamases (MBLs) are produced by many species of Gram-negative bacteria and confer resistance to carbapenems, cephalosporins, and penicillins except monobactams (Tada et al., 2019). New Delhi Metallo-beta-Lactamase 1 (NDM-1) is a plasmid-associated Ambler class B β-lactamase/carbapenemase that was first reported in clinical *E. coli* and *Klebsiella pneumoniae* isolates from an Indian patient in Sweden in 2008 (Yong et al., 2009). Subsequent cases of carbapenemase-producing isolates have since been reported in Britain, Australia, India, Russia, etc. (Yong et al., 2009; Kumarasamy et al., 2010; Bocanegra-Ibarias et al., 2017), and clinical isolates of *R. ornithinolytica* from urethral effluent, fester, and rectum samples have recently been found to produce this enzyme (Li et al., 2012; Khajuria et al., 2013; Zhou et al., 2013; Zheng et al., 2015; Paskova et al., 2018).

*R. ornithinolytica* infections are largely nosocomial and have rarely been reported in a healthy community (Seng et al., 2016). Nevertheless, the high rates of antimicrobial resistance in *R. ornithinolytica* isolates should be characterized in order to provide a basis for treating infections. To this end, we conducted a cross-sectoral study as part of the Sino-Swedish Integrated Multisectoral Partnership for Antibiotic Resistance Containment (IMPACT) in the Shandong Province in China using a One Health approach. The aim of this project is to study the relationship between the development of drug resistance in human (symbiotic and clinical), zoonotic, food, and environmental isolates of *R. ornithinolytica*. We identified NDM-1 and ESBL-producing *R. ornithinolytica* strains from healthy subjects and analyzed the drug resistance phenotypes and underlying mechanisms. To the best of our knowledge, this is the first report detailing the presence of an NDM-producing *R. ornithinolytica* strain in the human gut microbiota.

## Methods

### Bacterial Isolation and Identification

A total of 1,380 fecal samples were collected from healthy people in rural communities in July 2017 according to a previously described sampling procedure (Sun et al., 2018). Briefly, the samples were collected into ESwab tubes (Copan, Brescia, Italy) and stored at −80°C until cultivation. After thawing, the fecal samples were cultured on ChromID CARBA agar and ChromID ESBL agar plates (bioMérieux, Marcy l’Etoile, France) for 18 h at 37°C to respectively screen for the carbapenemase- and ESBL-producing *R. ornithinolytica* strains. The suspected *R. ornithinolytica* colonies identified based on color and morphology were picked and sub-cultured on CHROMagar Orientation agar (CHROMagar Company, Paris, France) overnight at 37°C. The resulting isolates were identified using MALDI-TOF MS and then confirmed by 16S rDNA sequence analysis against the bacterial 16S rDNA gene sequence in GenBank. The genomic average nucleotide identity (ANI) was calculated as described previously (Jiang et al., 2018).

### DNA Extraction and PCR

DNA was extracted from pure cultures of *R. ornithinolytica* using a Gentra Puregene Yeast/Bact. Kit (QIAGEN, Hilden, Germany), and the 16S rDNA sequences were amplified by PCR with primers designed using the Primer 5.0 software (3′-AGAGTTTGATCCTGGCTCAG-5′/3′-GGTTACCTTGTTAGGACTT-5′). The optimized cycling conditions were: initial denaturation at 94°C for 5 min followed by 30 cycles of amplification each with 94°C for 1 min, 52°C for 1 min, and 72°C for 1 min and final extension at 72°C for 5 min. The PCR product was detected by capillary electrophoresis as described previously (Zhang et al., 2019). The *bla*_NDM-1_, *bla*_CTX-M-3_, and *bla*_CTX-M-14_ genes were amplified by PCR using primers published previously (Shimizu et al., 2017; Chetri et al., 2019), and the products were confirmed by capillary electrophoresis followed by Sanger sequencing.

### Antimicrobial Resistance Test

To verify carbapenemase production by the isolates, resistance against imipenem, ertapenem, and/or meropenem were determined using the modified Hodge test. ESBL-production was verified using the double disk diffusion method with cefotaxime, ceftazidime, and/or clavulanic acid according to Clinical and Laboratory Standards Institute (CLSI, 2017).

### Antimicrobial Susceptibility Test

A total of 17 antibiotics belonging to 13 antimicrobial classes were tested, including cephalosporins (cefotaxime and ceftazidime), cephamycins (cefoxitin), β-lactam/β-lactamase inhibitor complexes (amoxicillin-clavulanate and piperacillin-tazobactam), carbapenems (imipenem and meropenem), penicillins (ampicillin), aminoglycosides (gentamicin and amikacin), fluoroquinolones (ciprofloxacin), folate metabolic pathway inhibitors (trimethoprim-sulfamethoxazole), tetracycline, chloramphenicols (florfenicol), colistin, furantoin, and tigecycline. The minimal inhibitory concentrations (MICs) of colistin and tigecycline were determined by the broth microdilution method and of other antibiotics using the agar dilution method. The results were interpreted according to CLSI (2017) and European Committee on Antimicrobial Susceptibility Testing (EUCAST) (version 8.1, 2018) guidelines. *E. coli* ATCC25922 was used as the quality control strain. Since clinical breakpoints of florfenicol are not available for Enterobacteriaceae in EUCAST or CLSI, the resistance breakpoint of >16 mg/L was selected based on the epidemiological cut-off values for the closely related *E. coli* and *Salmonella* spp. (Chi et al., 2019).

### Whole-Genome Sequencing

Whole-genome sequencing was performed on the extracted DNA by Sangon Biotech (Shanghai, China) using the Illumina HiSeq sequencing platform. The quality of the high-throughput sequence data was assessed by FastQC^1^. SPAdes 3.11.0 was used for assembling raw sequences (Hu et al., 2011). All draft genomes were deposited in the NCBI database under accession number VJYE00000000-VJYH00000000. Acquired antimicrobial resistance genes and plasmid replicons were identified using ResFinder 2.1 and PlasmidFinder 1.3^2^, respectively. The gene sequences surrounding *bla*_NDM-1_ and *bla*_CTX-M_ were annotated using RAST^3^ and Easyfig 2.2.3 (Chi et al., 2019). To find the core genes of the *R. ornithinolytica* genomes, Roary was used for SNPs analysis (Page et al., 2015). Maximum likelihood-based phylogenetic reconstruction was performed with RAxML version 8.2.10 (Stamatakis, 2014) and visualized with FastTree (Price et al., 2009).

### S1 Nuclease-Pulsed-Field Gel Electrophoresis and Southern Blotting

The location of *bla*_NDM-1_, *bla*_CTX-M-3_, and *bla*_CTX-M-14_ on the plasmids was validated by S1-PFGE and southern blotting. Briefly, the isolates were embedded in 10 g/L Seakem Gold gel, digested with endonuclease S1 nuclease (TakaRa, Dalian, China), and subjected to pulsed-field gel electrophoresis (Parameters: 14°C, voltage 6 V/cm, electric field angle 120°, conversion time 2.16–63.8 s, and electrophoresis 16 h). The DNA fragments were transferred horizontally to a nylon membrane (Millipore, USA), and hybridized with three digoxin-labeled probes obtained by PCR amplification (Yu et al., 2002) and the Dig High Prime DNA Labeling and Detection Starter Kit (Roche Diagnostics). The genomic DNA of *Salmonella enterica* serovar Braenderup H9812 strain cut with *Xba*I was used as the DNA marker.

### Conjugation Assay

The horizontal transferability of *bla*_NDM-1_, *bla*_CTX-M-3_, and *bla*_CTX-M-14_ were examined using conjugation assay using *E. coli* J53 (azide-resistant) or *E. coli* EC600 (rifampicin-resistant) as the recipient strains. The recipient bacteria and the target single colony were inoculated into 2 ml LB liquid medium and cultured at 37°C with constant shaking for about 6.5 h till the logarithmic growth phase. The recipient and donor bacteria culture broths were mixed at 1:2 v/v (200 μl and 400 μl), inoculated in 2 ml LB liquid medium, and incubated at 37°C for 12 h. The following day, 100 μl of the culture broth was uniformly spread on sodium azide-resistant agar containing cefotaxime (imipenem) and rifampicin and cultured at 37°C for 18 h. The single colonies were picked and purified overnight. Conjugants were identified by the presence of antibiotic resistance genes that were detected using PCR as described.

## Results

### Multidrug-Resistant *Raoultella ornithinolytica* Strains Were Isolated From the Human Fecal Samples

**Figure 1:**
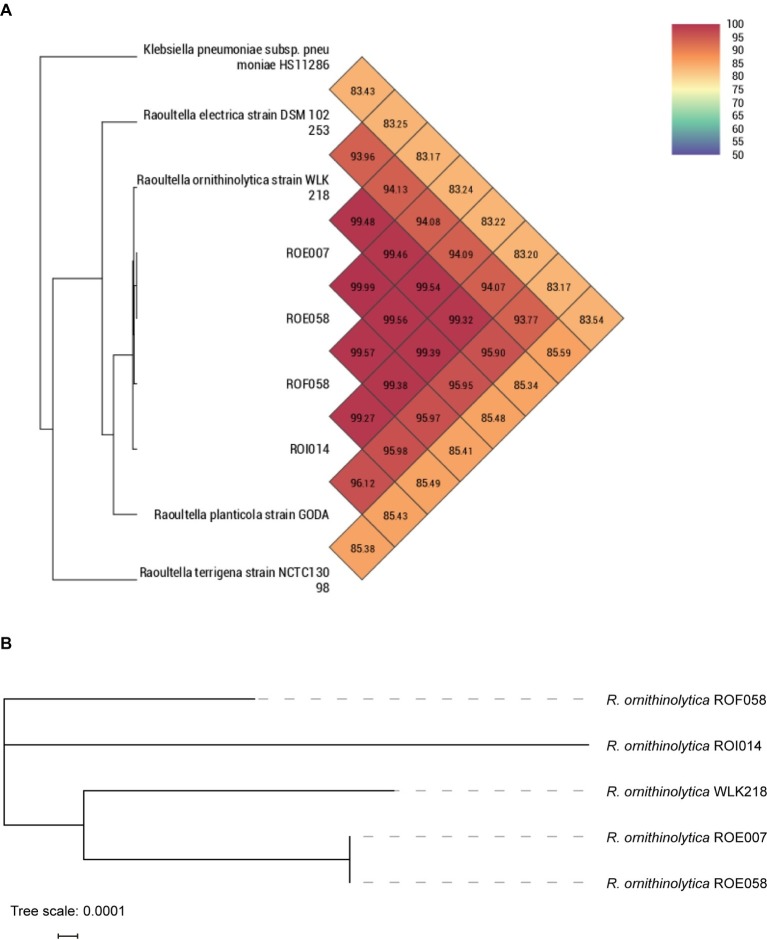
Phylogenetic comparison depicting the relationship of *R. ornithinolytica* strain isolated in this study with other *Raoultella* species. **(A)** ANI analysis of *R. ornithinolytica* isolates with other *Raoultella* species. **(B)** Core genome phylogeny for the *R. ornithinolytica* isolates.

One carbapenemase-producing (ROF058) and three ESBL-producing (ROE007, ROE058, and ROI014) *R. ornithinolytica* strains were isolated from 1,380 fecal samples (Table 1) and confirmed by MALDI-TOF MS, 16S rDNA sequencing, and ANI analysis (Figure 1A). Phylogenetic analysis revealed that isolates ROE007 and ROE058 are clonally related although they were recovered from different villagers in the same natural village (Figure 1B). The *bla*_NDM-1_, *bla*_CTX-M-3_, and *bla*_CTX-M-14_ genes were subsequently identified in all the strains, and multidrug resistance was confirmed by antimicrobial susceptibility tests. ROF058 was resistant to 12 antibiotics other than carbapenem, but sensitive to amikacin, tigecycline, furantoin, colistin, and trimethoprim-sulfamethoxazole. The resistance profiles of the three ESBL-producing isolates were similar, and all were resistant to gentamicin, tetracycline, cefotaxime, trimethoprim-sulfamethoxazole, and ampicillin. Furthermore, all trans-conjugants exhibited MDR phenotypes similar to the donor strain (Table 1).

**Table 1:** The minimum inhibitory concentrations of tested antimicrobial agents against the *R. ornithinolytica* isolates and the respective conjugants.

Isolates	MICs (μg/ml)																
	CTX	CAZ	CFX	AMC	PTZ	IMP	MEM	AMP	GEN	AMI	CIP	SXT	TET	FFN	COL	NIT	TGC
ROE007	**32**	2	2	8	4	0.032	0.032	**>128**	**16**	0.5	1	**8**	**128**	2	2	16	0.5
ROE058	**16**	2	**4**	8	2	0.25	0.032	**>128**	**16**	2	0.5	**8**	**128**	**4**	2	16	0.5
ROI014	**8**	1	≤1	4	0.5	0.064	0.008	**>128**	**32**	1	0.25	**8**	**32**	2	2	8	0.5
R0F058	**>128**	**128**	**>128**	**>32**	**>128**	**64**	**32**	**>128**	**>128**	2	**64**	0.125	**>128**	**8**	2	32	0.5
CR0E007-J53	**32**	2	**4**	4	2	0.032	0.032	**>128**	**32**	1	0.5	0.5	**>128**	**4**	2	8	0.5
CR0E058-J53	**32**	2	**4**	4	2	0.032	0.032	**>128**	**32**	1	0.5	0.25	**>128**	**8**	2	8	0.5
CR0I014-J53	**16**	2	2	4	2	0.032	0.016	**>128**	**16**	2	0.5	0.5	**>128**	2	2	8	0.5
CR0F058-EC600	**>128**	**>128**	**>128**	4	**128**	2	**16**	**>128**	**32**	2	0.5	1	**>128**	**4**	2	16	0.5
J53	1	0.125	**4**	4	1	1	4	3	1	4	0.125	0.125	**32**	**8**	1	4	0.25
EC600	0.5	3	2	2	16	0.5	2	3	1	1	0.25	0.125	**32**	2	1	4	0.25

*CTX, cefotaxime; CAZ, ceftazidime; CFX, cefoxitin; AMC, amoxicillin-clavulanic acid; PTZ, piperacillin-tazobactam; IMP, imipenem; MEM, meropenem; AMP, ampicillin; CIP, ciprofloxacin; SXT, trimethoprim-sulfamethoxazole; TET, tetracycline; FFN, florfenicol; COL, colistin; NIT, furantoin; TGC, tigecycline. The bold font indicates resistance to the tested antimicrobial agent*.

### The *Raoultella ornithinolytica* Strains Harbor Multidrug-Resistant Genes

**Figure 2:**
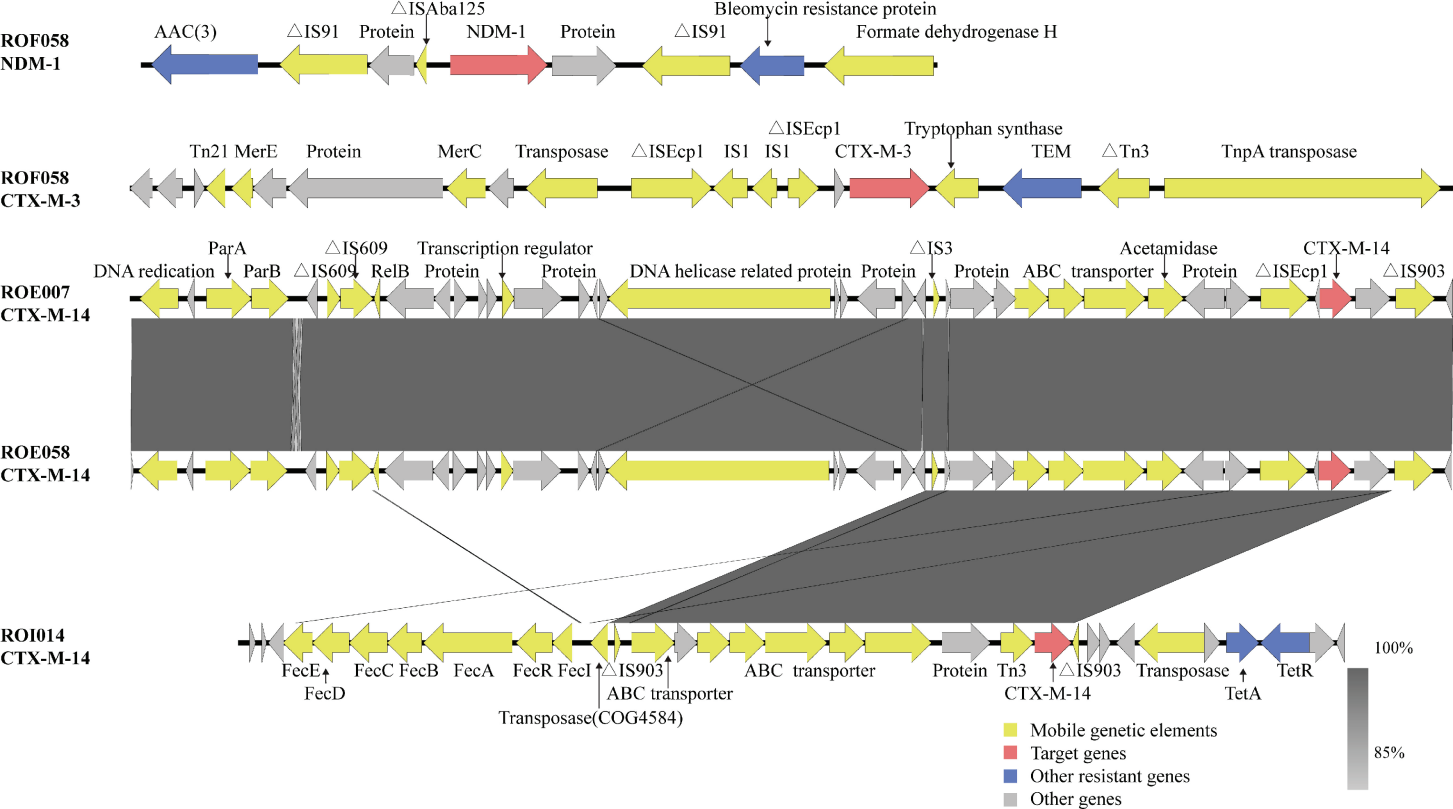
Genetic analysis of the blaNDM-1 and blaCTX-M genes. Arrows represent direction of transcription. Red open reading frames (ORFs) indicate NDM-1/CTX-M, blue ORFs drug resistance-associated proteins, yellow ORFs mobile genetic elements, and gray ORFs other proteins or proteins of unknown function.

MDR was defined as acquired non-susceptibility to at least one agent in three or more antimicrobial categories, and XDR was defined as non-susceptibility to at least one agent in all but two or fewer antimicrobial categories (Magiorakos et al., 2012). Whole genome sequencing showed that ROF058 is an extensively drug-resistant (XDR) strain, while ROE007, ROE058, and ROI014 are MDR strains (Table 1). In addition to the carbapenemase-encoding *bla*_NDM-1_, ROF058 also carries genes encoding for other ESBLs (*bla*_CTX-M-3_, *bla*_OXA-1_, *bla*_TEM-1B_, and *bla*_DHA-1_), as well as resistance factors against, aminoglycosides [*aac*(*3*)*-IId*], rifampicins (*arr-3*), chloramphenicols (*catB3*), tetracyclines [*tet*(*D*)], quinolones [*qnrB4 and aac*(*6*′)*-Ib-cr*], fosfomycin (*fosA*), and sulfonamides (*sul1*) (Figure 2). The remaining isolates harbored genes encoding the ESBLs (*bla*_CTX-M-14_), quinolones (*qnrS1*), aminoglycosides [*aac*(*3*)*-IId*], sulfonamides (*sul1*), tetracyclines [*tet*(*A*)], sulfanilamides (*dfrA1*), and fosfomycins (*fosA*) resistance genes.

### The Multidrug-Resistant Genes Are Located on Mobile Genetic Elements

**Figure 3:**
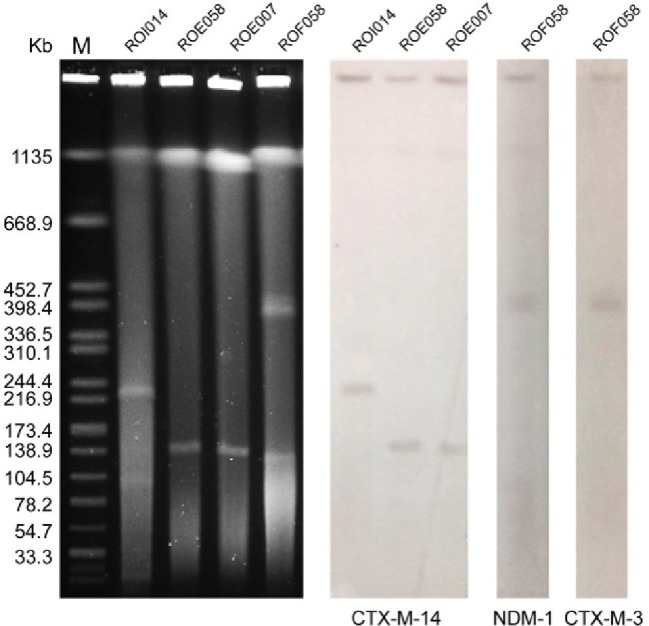
S1-PFGE analysis of the *R. ornithinolytica* isolates and southern hybridization. Lane M, molecular weight marker Salmonella Braenderup H9812; Line 1, strain ROI014; Line 2, ROEO58; Line 3, strain ROE007; Line 4, strain ROF058; Line 5, CTX-M-14 hybridization for ROI014; Line 6, CTX-M-14 hybridization for ROE058; Line 7, CTX-M-14 hybridization for ROE007; Line 8, NDM-1 hybridization for ROFO58; Line 9, CTX-M-3 hybridization for ROFO58.

Whole genome sequencing of strain ROF058 generated 5,747,150 clean reads, which were then assembled to 209 contigs with a GC content of 51.38%; WGS of strain R0E007 generated 5,214,213 clean reads, which were then assembled to 203 contigs with a GC content of 49.36%; WGS of strain ROE058 generated 5,235,762 clean reads, which were then assembled to 199 contigs with a GC content of 50.28%; WGS of strain ROI014 generated 5,510,178 clean reads, which were then assembled to 215 contigs with a GC content of 54.66%. The plasmids in the different isolates were identified using the Center for Genomic Epidemiology program (Luo et al., 2017), which showed that ROF058 harbored the IncFIB(K)-type plasmid, ROE007 and ROE058 contained IncFIB(K) and IncFII-type plasmids, and ROI014 carried IncFIB(K) and IncFII plasmids. In addition, S1-PFGE and southern blot hybridization revealed three different plasmids in the *R. ornithinolytica* isolates ranging from 104.5 to 398.4 kb (Figure 3). ROFO58 contained the larger plasmid (336.5–398.4 kb) harboring the *bla*_NDM-1_ and *bla*_CTX-M-3_ genes. R0E007 and ROE058 had identical plasmid profiles, and the *bla*_CTX-M-14_ gene was located on a 138.9 kb plasmid in both isolates. The *bla*_CTX-M-14_ gene in ROI014 was located on a 216.9–244.4 kb plasmid. Furthermore, the *bla*_NDM-1_ and *bla*_CTX-M-3_ genes could be transferred from each of the isolates into recipient *E. coli* strains *via* conjugation (Table 1), which was confirmed by PCR (data not shown).

## Discussion

Intermittent but lethal nosocomial infections by *R. ornithinolytica* have been increasingly reported in the past decade (De Petris and Ruffini, 2018), and the advent of techniques like MALDI-TOF MS has enabled identification of new isolates (De Jong et al., 2013; Seng et al., 2016). Notable reports of *R. ornithinolytica* infections include sepsis in a patient with mitochondrial disease in Spain (Sanchez-Codez et al., 2019), urinary tract infection in a pediatric patient in Turkey (Buyukcam et al., 2019), and ventilator associated pneumonia in two immunocompetent trauma patients in USA (Van Cleve et al., 2018). *R. ornithinolytica* has also been implicated in joint infections and appendicitis in the clinical setting (Beye et al., 2018; Hajjar et al., 2018). In addition, MDR strains of this pathogen have emerged in recent years, primarily due to the spread of the resistance genes. The first NDM-1-producing *R. ornithinolytica* strain was isolated from the pus of hospitalized patients in India in 2013 (Khajuria et al., 2013) and was followed by reports of resistant *R. ornithinolytica* in USA, China, India, Spain, Korea, Bangladesh, and Brazil. In addition, some resistant strains have also been isolated from animals, vegetables, wastewater, and river water. The major drug resistance genes identified in these isolates include *bla*_NDM-1_, *bla*_KPC-2_, *mcr-1*, *bla*_CTX-M-15_, *bla*_IMP-4_, and *bla*_OXA-1_ (Zhou et al., 2014; Hasan et al., 2015; Zheng et al., 2015; Hernandez-Garcia et al., 2018; Miao et al., 2018; Yoon et al., 2018; Carramaschi et al., 2019; Wang et al., 2019). However, little is known regarding the carbapenemase- and ESBL-producing *R. ornithinolytica* strains. To the best of our knowledge, this is the first report on resistant *R. ornithinolytica* isolated from human gut microbiota.

Studies show that bacterial strains producing NDM-1 are resistant to most of the antibiotics used clinically and are only sensitive to a few such as colistin and tigecycline (Khan et al., 2017). However, Wang et al. (2018) described a strain of *K. pneumoniae* isolated from both humans and animals that was positive for the NDM-1 gene and showed resistance to polymyxin and colistin (Wang et al., 2018). Consistent with this, our drug susceptibility test showed that ROFO58 was resistant to cefotaxime, ceftazidime, gentamicin, tetracycline, and ciprofloxacin. *R. ornithinolytica* is naturally resistant to aminopenicillin due to the presence of the chromosomal class A β-lactamase gene (Vasaikar et al., 2017), and all isolates did exhibit resistance to ampicillin. The presence of drug resistance genes strongly correlated with resistant phenotypes. Therefore, the WGS approach can rapidly detect antibiotic resistance genes that have been annotated and predict drug-resistance in a particular isolate. Although all trans-conjugants showed an MDR phenotype similar to that of the donor strain in our study, the NDM-1-producing isolate had lower resistance to gentamicin and were sensitive to imipenem, ciprofloxacin, and amoxicillin-clavulanic acid. This could be due to factors other than the resistance genes. For example, studies have shown that the same resistance gene confers different susceptibility patterns when under the control of different promoters (Zhou et al., 2019).

A previous report showed that the *bla*_NDM-1_ gene in Enterobacteriaceae is located on a rapidly transferable 50–200 kb plasmid belonging to several incompatibility groups such as IncL/M, IncHI1, IncFIIs, IncF, or untypable (Ahmad et al., 2018). In this study, the plasmid carrying *bla*_NDM-1_ was larger than 300 kb and of type IncFIB. Furthermore, this plasmid could be transferred from R0F058 to *E. coli* EC600 but not to *E. coli* J53. We surmised therefore that the plasmid was rare and selectively transferred. Epidemiological and genetic studies have shown that plasmid ligation and transposition of mobile genetic elements play an important role in the horizontal transmission of *bla*_NDM-1_ (Khajuria et al., 2013). BLAST analysis showed the presence of the aminoglycoside acetyltransferase gene [*aac(3)*] and bleomycin resistance gene upstream and downstream of *bla*_NDM-1_, respectively. There were two insertion sequences (ΔIS*91*) flanking both resistance genes, indicating its important role in the spread of *bla*_NDM-1_ and other resistant genes. In addition, a remnant of ΔIS*Aba125* was located upstream of *bla*_NDM-1_ (Figure 2). A previous study has shown that partial IS*Aba125* is a promoter of the *bla*_NDM-1_ gene (Poirel et al., 2011). The *bla*_NDM-1_ and *bla*_CTX-M-3_ genes were detected in the ROF058 trans-conjugant, indicating that *bla*_NDM-1_ can be co-transferred with *bla*_CTX-M-3_ and across species, and thereby disseminate drug resistance. Although clinically important bacteria such as *Enterobacter cloacae* and *Klebsiella pneumoniae* produce both NDM-1 and CTX-M-3 (Rubin et al., 2014; Hu et al., 2016), we have shown the co-existence of *bla*_NDM-1_ and *bla*_CTX-M-3_ in one plasmid for the first time.

The isolates in our study carried the *bla*_CTX-M-3_ and *bla*_CTX-M-14_ genes, which is similar to the CTX-M types of ESBL-producing *K. pneumoniae* isolated from environmental samples (Chi et al., 2019). This likely indicates spread of resistant bacteria between human hosts and the environment. The *bla*_CTX-M_ genes are usually present on the IncF, IncI, IncN, IncHI2, IncL/M, and IncK plasmid types (Zhang et al., 2016). WGS analysis showed that three ESBL-producing *R. ornithinolytica* isolates in our study carry the IncFIC plasmid, along with a ΔIS*Ecp1* sequence upstream of *bla*_CTX-M-3_ in ROF058, and *bla*_CTX-M-14_ in ROE007 and ROE058. A study showed that 97.6% of the *bla*_CTX-M_ genes have an upstream ΔIS*Ecp1* sequence (Adamski et al., 2015), which plays an important role in the transposition of *bla*_CTX-M_ and other ESBL genes (Decano et al., 2019). R0E007 and ROE058 isolated from the same village showed an identical sequence flanking *bla*_CTX–M–14_, i.e., ΔIS*Ecp1*-*bla*_CTX-M-14_-ΔIS*903*, which could be the result of either the clonal expansion of the original host bacteria or lateral transfer of the genetic elements. A linear *fecE-fecD-fecC-fecB-fecA-fecR-fecI* ferric citrate genes were detected upstream of *bla*_CTX-M-14_ in ROI014, which is very similar to that reported in an environmental *K. pneumoniae* isolate (accession no. KF914891) from the same area (Chi et al., 2019). These findings strongly indicate the spread of drug-resistance genes between *K. pneumoniae* and *R. ornithinolytica*.

To summarize, we characterized *R. ornithinolytica* isolates from healthy human feces in terms of their antibiotic susceptibility, drug resistance genes, and the transfer mechanism of the mobile genetic elements. The strains co-producing NDM-1 and CTX-M can confer higher levels of resistance to multiple antibiotics and can transfer the genes to other strains *via* plasmids by conjugation. Therefore, the patterns of antibiotic resistance and transmission should be closely monitored for *R. ornithinolytica*, especially in healthy individuals.

## Data Availability Statement

The datasets generated for this study can be found in the National Center for Biotechnology Information, VJYE00000000-VJYH00000000, https://submit.ncbi.nlm.nih.gov/.

## Ethics Statement

The studies involving human participants were reviewed and approved by Ethics Committee for Shandong Center for Disease Control and Prevention; Shandong Center for Disease Control and Prevention. The patients/participants provided their written informed consent to participate in this study.

## Author Contributions

SW and LX contributed to perform the experiments, data analysis, and manuscript writing. XC and YL contributed to perform the experiments. XC, ZK, PH, HX, ZB, and BZ contributed to the sample collection and data analysis. BZ, ZB, and HX contributed to conceive and design the experiments and reviewed the article.

### Conflict of Interest

The authors declare that the research was conducted in the absence of any commercial or financial relationships that could be construed as a potential conflict of interest.

## Footnotes

^1^http://www.bioinformatics.babraham.ac.uk/projects/fastqc/

^2^https://cge.cbs.dtu.dk

^3^http://rast.nmpdr.org/rast.cgi
